# *Klebsiella pneumoniae* factors enhancing bacteremia have distinct contributions to macrophage-mediated, oxidative, and nitrosative stress resistance

**DOI:** 10.1128/iai.00739-25

**Published:** 2026-02-23

**Authors:** Alexis E. Wilcox, Catherine J. Andres, Michael A. Bachman, Caitlyn L. Holmes

**Affiliations:** 1 Department of Microbiology and Immunology, University of Minnesota Medical School, Minneapolis, Minnesota, USA; 2 Department of Pathology, University of Michigan Medical School, Ann Arbor, Michigan, USA; 3 Center for Immunology, University of Minnesota Medical School, Minneapolis, Minnesota, USA

**Keywords:** gram-negative bacteria, *Klebsiella*, innate immunity, macrophages, stress response, bacteremia, bloodstream infections, oxidative stress, nitrosative stress

## Abstract

*Klebsiella pneumoniae* is a Gram-negative species that is a leading cause of hospital-associated infections. Such infections can result in bacteria, when bacteria disseminate to the bloodstream and colonize filtering organs. While interactions between alveolar macrophages and *K. pneumoniae* have been described in the context of pneumonia, less is known about interactions between *K. pneumoniae* and monocyte-derived macrophages, which are present during bacteremia across tissues. The antibacterial stress mechanisms used by innate immune cells and the genes *K. pneumoniae* utilizes to resist macrophage-mediated killing are poorly understood in the context of bacteremia. Here, we investigated the role of capsule, hypermucoviscosity, and 53 previously identified *K. pneumoniae* bacteremia fitness factors for their role in resistance against oxidative, nitrosative, and macrophage-mediated stress. Increased *K. pneumoniae* hypermucoviscosity correlated with lower uptake by macrophages, but the polysaccharide capsule did not enhance intracellular fitness. About 60% of *K. pneumoniae* bacteremia fitness factors enhanced resistance to oxidative, nitrosative, or macrophage-mediated stress, but often did so in distinct manners. Some factors were involved in resistance to a single stressor, while other factors were linked to multiple stressors. DNA repair mechanisms were important for resisting multiple stressors, while transcriptional regulator function was linked to nitrosative stress. Additionally, a factor’s ability to enhance nitrosative stress resistance was significantly correlated with intracellular fitness and fitness in the spleen during infection. These findings provide new insights into the relationship between innate immunity and *K. pneumoniae*, furthering our understanding of the strategies employed by *K. pneumoniae* to withstand stress during bacteremia.

*Klebsiella pneumoniae* is a Gram-negative pathogen and a leading cause of bacteremia, the presence of bacteria in the bloodstream (1–3). Bacteremia is particularly dangerous as it can initiate sepsis and is linked to high mortality rates, especially if the causative pathogen is resistant to antimicrobials (4–6). *K. pneumoniae* has been repeatedly classified as a pathogen of urgent concern due to increasing rates of antimicrobial resistance, yet little is known about how *K. pneumoniae* resists host defenses during systemic infections such as bacteremia (7–9). The pathogenesis of Gram-negative bacteremia occurs in three phases (10). First, bacteria invade an initial site, commonly the lung or gut in *K. pneumoniae* infection. Second, bacteria disseminate into the bloodstream using a variety of species- and site-specific mechanisms (11, 12). Third, bacteria survive in the bloodstream by colonizing filtering organs like the spleen and liver and must avoid immune clearance mechanisms within these tissues (13–17). As rates of antimicrobial resistance rise, understanding host-pathogen interactions that allow *K. pneumoniae* to resist host-mediated stress in the bloodstream will illuminate new targets for future therapies and allow insight into the pathogenesis of this important species (18, 19).

Macrophages are a first-line member of the innate immune response and play a pivotal role during *K. pneumoniae* infection. Tissue-resident alveolar macrophages assist in the initial recognition, phagocytosis, and clearance of *K. pneumoniae* in the lung (20). Monocyte-derived macrophages home to sites of inflammation and are also important in *K. pneumoniae* infection, as depletion of this subset leads to a worse outcome in murine disease models (21, 22). While the importance of macrophage-mediated stress to the trajectory of pneumonia has been well established, it remains unknown how *K. pneumoniae* itself combats this prominent host defense.

Our work has identified that *K. pneumoniae* resistance to oxidative stress is important during bacteremia (16). The phagocyte NADPH oxidase Nox2 substantially contributes to *K. pneumoniae* infection by controlling bacterial replication in the lung and preventing certain modes of dissemination to the blood (11, 16, 23). The *K. pneumoniae* factor SspA, a regulator of the stringent starvation response, protects the bacteria from both *in vitro* oxidative stress and Nox2-dependent stress during bacteremia. PdxA, a member of the vitamin B_6_ biosynthesis pathway, enhances *K. pneumoniae* resistance against *in vitro* oxidative stress but likely protects against additional stressors *in vivo* (16). GmhB, a bacteremia fitness factor involved in inner core lipopolysaccharide (LPS) biosynthesis, is dispensable for oxidative stress resistance *in vitro* but influences *K. pneumoniae* lung dissemination patterns, which are dependent on Nox2 (15). Thus, we hypothesized that macrophages present across sites of infection are a source of oxidative and other stresses that *K. pneumoniae* must overcome to cause bacteremia.

To define the overlap between *K. pneumoniae* genes that enhance bacteremia and those that increase resistance to prominent forms of stress elicited by the innate immune system, we leveraged our existing set of *K. pneumoniae* bacteremia fitness factors and assessed the ability of each to protect the bacteria from oxidative, nitrosative, and macrophage-mediated stress. This work is the first to define that *K. pneumoniae* bacteremia fitness factors often have distinct contributions to resisting multiple forms of stress. Our study reveals interactions between *K. pneumoniae* and common forms of innate immune stress, furthering insight into host-pathogen interactions during bacteremia.

## RESULTS

### Using gentamicin protection assays to detect *K. pneumoniae* factors influencing intracellular fitness

To investigate *K. pneumoniae* resistance to macrophage-mediated stress, we first needed to establish appropriate conditions for high-throughput gentamicin protection assays. We aimed to select conditions that maximized KPPR1 uptake but minimized the ratio of bacteria to cells and contact time between the two, as these variables may initiate confounding stress responses such as cell death. First, we incubated macrophages with an increasing ratio of KPPR1-chromoGFP, a strain with constitutive chromosomal expression of GFP, ranging from a multiplicity of infection (MOI) of 1–50 (Fig. S1A). After 1 h of contact time, extracellular KPPR1 was killed with gentamicin, cells were fixed, and fluorescent microscopy was used to visualize the amount of KPPR1 associated with macrophages. As expected, an increasing MOI of *K. pneumoniae* increased the percentage of cells associated with KPPR1-chromoGFP. Next, we varied the contact time between KPPR1-chromoGFP and macrophages between 30 min and 4 h. There was a significant increase in association at 1 h compared to 30 min, but the percentage of infected macrophages was similar past this time point (Fig. S1B). Finally, KPPR1-chromoGFP was incubated with or without active mouse serum prior to infection, which did not influence the abundance of *Kp*-associated cells (Fig. S1C). Thus, in downstream experiments, we used an MOI of 10, a contact time of 1 h, and did not incubate bacteria with serum prior to infection (Fig. S1D i to iii). Macrophages infected with this approach had normal morphology at the experiment end point.

### Multiple *K. pneumoniae* bacteremia fitness factors influence resistance to macrophage-mediated intracellular stress

Next, we assessed how *K. pneumoniae* bacteremia fitness factors influenced interactions between KPPR1 and macrophages. Transposon mutants in 52 of the 58 genes identified in our previous study as contributing to bacteremia fitness (16) were available within an arrayed library (24), and we had access to a previously constructed Δ*arcA* mutant (25). The correct chromosomal location of the transposon was verified for each mutant by PCR (Materials and Methods), and mutants were grouped into broad functional categories informed by KEGG orthology (26). For each strain, uptake and susceptibility to macrophage-mediated stress (or intracellular stress) were assessed with gentamicin protection assays.

Minimal wild-type KPPR1 was taken up by macrophages (~7% of the input was detected intracellularly on average, Table S1), yet intracellular *K. pneumoniae* was observed in every trial. While multiple *K. pneumoniae* factors appeared to influence uptake, some of the subtle differences were likely attributable to slight variations in MOI between strains (Fig. S2; Table S1). Mutations in the *arn* operon led to significantly higher uptake, while mutations in *csrD, crp*, and *pitA* led to lower uptake (Fig. S2). These patterns correlated with levels of hypermucoviscosity (HMV) as the *arn* genes had substantially lower HMV, and *csrD, crp*, and *pitA* had higher HMV (Fig. S3, [24]). The connections between HMV and uptake have been previously described (27–29) and indicated that the assay and mutants were exhibiting expected behavior. However, there were exceptions to this trend. The *polA* mutant exhibited significantly higher HMV than KPPR1 (Fig. S3A), yet also had higher uptake (Fig. S2A). Thus, HMV is linked to macrophage uptake but does not exclusively predict this interaction.

**FIG 1:**
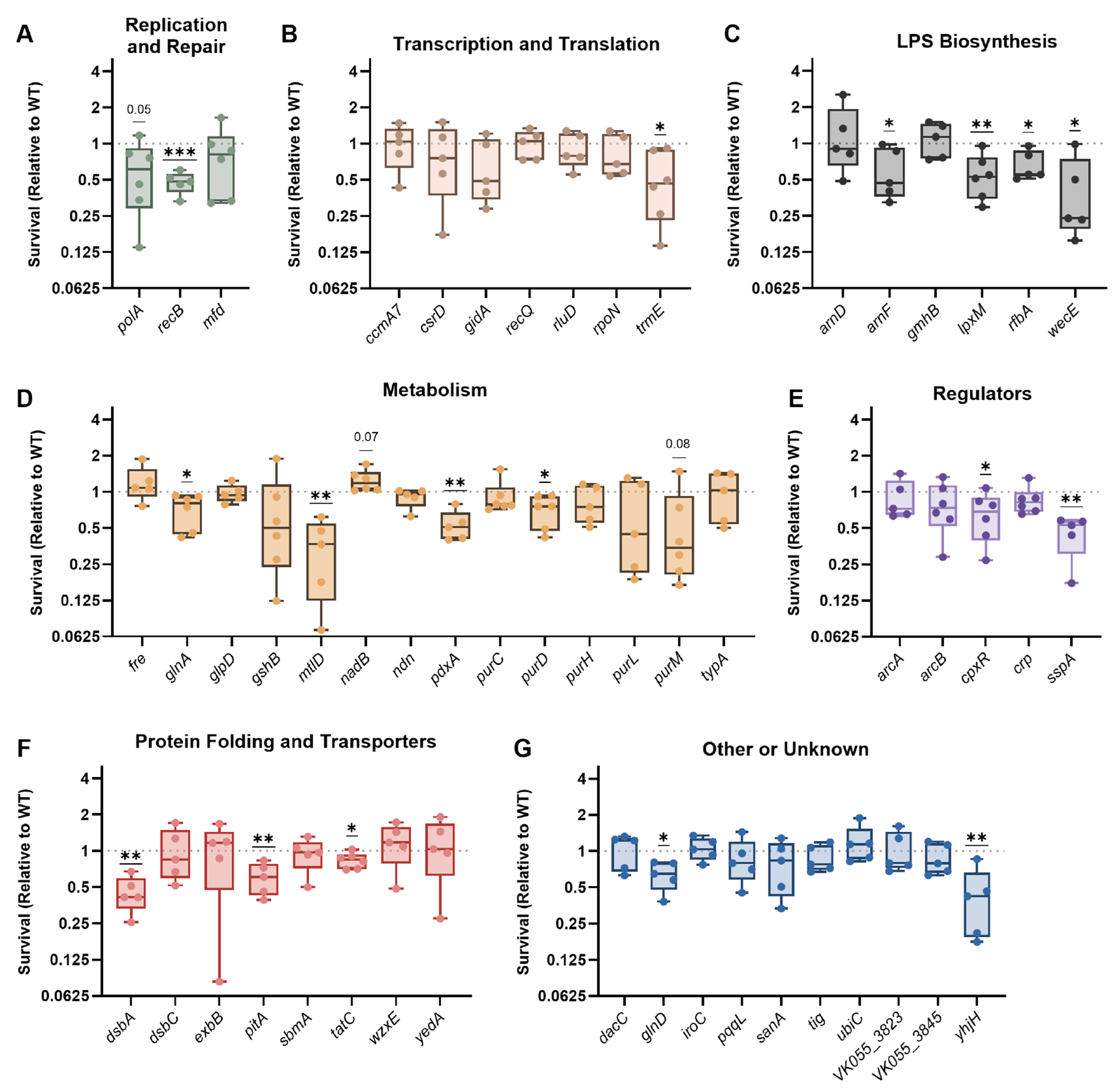
*K. pneumoniae* utilizes a variety of bacteremia fitness genes to resist macrophage-mediated stress. Bone-marrow-derived macrophages were infected with wild-type KPPR1 or transposon mutants with insertions in factors enhancing fitness in the spleen during bacteremia. Mutants were are displayed in groups by their predicted function, including (A) Replication and Repair, (B) Transcription and Translation, (C) LPS Biosynthesis, (D) Metabolism, (E) Regulators, (F) Protein Folding and Translocation, or (G) Other or Unknown functions. After 1 h of infection, extracellular bacteria were killed with gentamicin treatment, cells were lysed, and intracellular *K. pneumoniae* was enumerated to quantify bacterial uptake (T0). A separate subset of wells was incubated for an additional 4 h, treated with gentamicin, lysed, and intracellular *K. pneumoniae* was enumerated to quantify bacterial survival (T4). The percent survival of each strain was calculated as (CFU T4/CFU T0) × 100. Survival (Relative to WT) was calculated by dividing the percent survival of each mutant by the percent survival of WT KPPR1 within the same assay. All experiments were performed in four to six independent trials; *P < 0.05, **P < 0.01, and ***P < 0.001 by a one-sample *t*-test with a hypothetical value of 1, *P* < 0.10 are indicated in text, and other comparisons were considered not significant. In all, *y*-axis data are displayed as a log_2_ fold change; box plot lines display the 25th, 50th, and 75th percentile values, whiskers indicate the minimum and maximum values, and points represent values from individual trials.

Regarding intracellular survival, wild-type KPPR1 experienced an average of 42% survival within bone-marrow-derived macrophages (BMDMs) after 4 h (Table S1). Of 53 mutants, 17 had significantly lower survival compared to wild type (Fig. 1A through G) with an additional 2 genes experiencing fitness defects that did not reach statistical significance (*polA: P* = 0.05 and *purM: P* = 0.08, Fig. 1A and D). Each functional genetic category had at least one mutant with decreased intracellular fitness, but susceptibility to macrophage-mediated stress was particularly pronounced in the LPS Biosynthesis category, where four of six mutants experienced loss of intracellular fitness (Fig. 1C). This is not surprising since interactions between LPS and macrophages are well established. For example, the LPS core and O-antigen regions protect the bacterial membrane from multiple environmental stressors, many of which are similar to the types of stress that may be encountered in the intracellular space, such as antimicrobial peptides. Of 14 genes, 4 genes in the Metabolism category were important for *K. pneumoniae* intracellular survival, perhaps indicating a need for metabolic flexibility within an intracellular niche.

**FIG 2:**
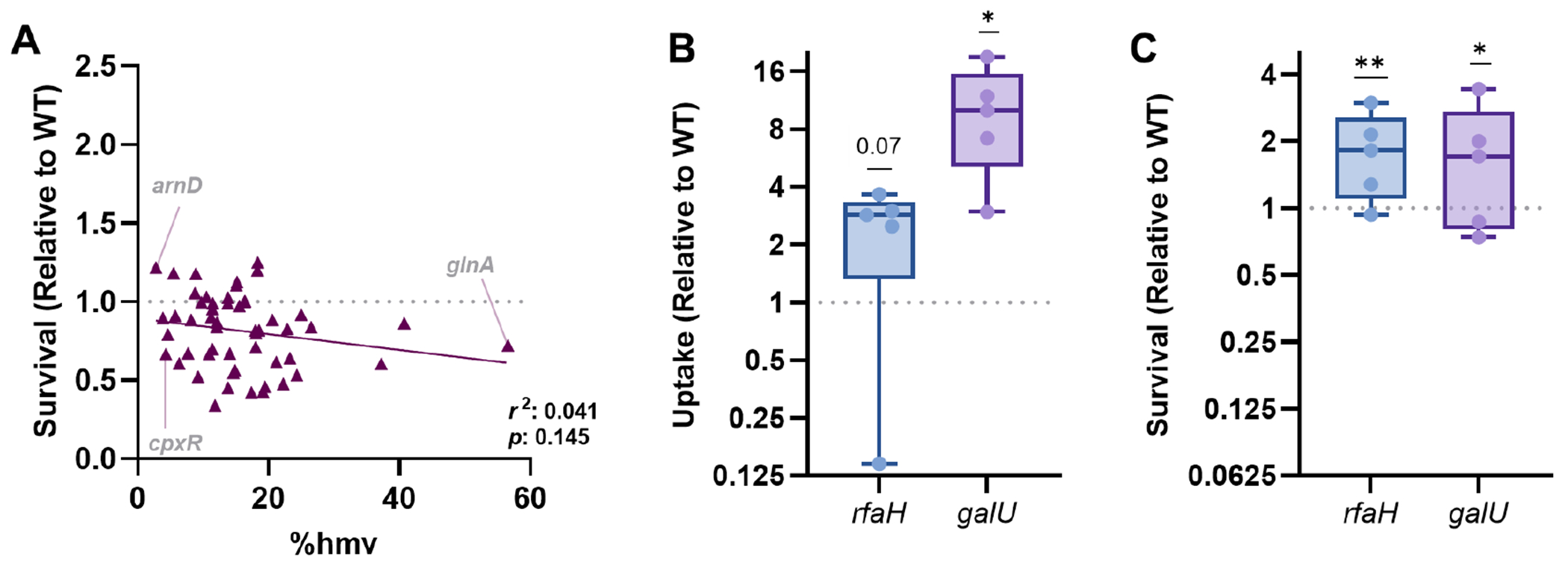
*K. pneumoniae* capsule is dispensable for intracellular survival. (A) The correlation between HMV and survival within macrophages was assessed using a Pearson’s coefficient (*r*^2^), and *P*-values are indicated. The gray text indicates mutants described in the main text. Acapsular KPPR1 strains Δ*rfaH* and Δ*galU* were assessed for either (B) uptake or (C) intracellular survival using gentamicin protection assays for bone marrow-derived macrophages. In (B), uptake was calculated as CFU/mL at T0. In (C), the percent survival of each strain was calculated as (CFU T4/CFU T0) × 100. Uptake or survival (Relative to WT) was calculated by dividing the percent (B) uptake or (C) survival of each mutant by that of WT KPPR1 within the same assay. Experiments were performed in five independent trials; *P < 0.05 and **P < 0.01 by a one-sample *t*-test with a hypothetical value of 1. Box plot lines display the 25th, 50th, and 75th percentile values, whiskers indicate the minimum and maximum values, and points represent values from individual trials. For all, *y*-axis data are displayed as a log_2_ fold change.

Originally, we hypothesized that mutants with elevated HMV would have higher resistance to intracellular threats. However, this was not the case as HMV was not significantly correlated with intracellular survival (Fig. 2A). While some mutants with lower HMV had lower intracellular fitness (e.g., *cpxR;* Fig. 1E and 2A), lower HMV often led to no alterations in survival after macrophage-mediated stress (e.g., *arnD;* Fig. 1C and 2A). In contrast, strains with mutations that resulted in elevated HMV often had lower intracellular survival compared to wild-type KPPR1 (e.g., *glnA;* Fig. 1D and 2A). HMV is a function of capsule polysaccharide chain length and diversity on the bacterial surface (27, 30), so we next tested whether capsular polysaccharide was required for intracellular survival. Using two previously characterized acapsular KPPR1 strains (15, 24, 31), Δ*rfaH* and Δ*galU*, we confirmed that these mutants had higher uptake by macrophages compared to wild-type KPPR1 (Fig. 2B). Surprisingly, the acapsular strains experienced no intracellular fitness defects and instead demonstrated a substantial survival advantage over wild-type KPPR1 (Fig. 2C). These results reveal that while HMV and capsule are important for *K. pneumoniae* to resist uptake by macrophages, they likely do not play a role in enhancing intracellular fitness. In contrast, capsules may convey a disadvantage for *K. pneumoniae* intracellular fitness.

### *K. pneumoniae* uses diverse strategies to resist oxidative and nitrosative stress

Two major forms of stress used by innate immune cells to kill pathogens are oxidative and nitrosative stress. To assess whether *K. pneumoniae* bacteremia fitness factors were associated with resistance to one, both, or neither form of stress, each transposon mutant was exposed to oxidative stress (elicited by hydrogen peroxide) or nitrosative stress (elicited by DETA NONOate), and the percent survival relative to wild-type KPPR1 was calculated.

**FIG 3:**
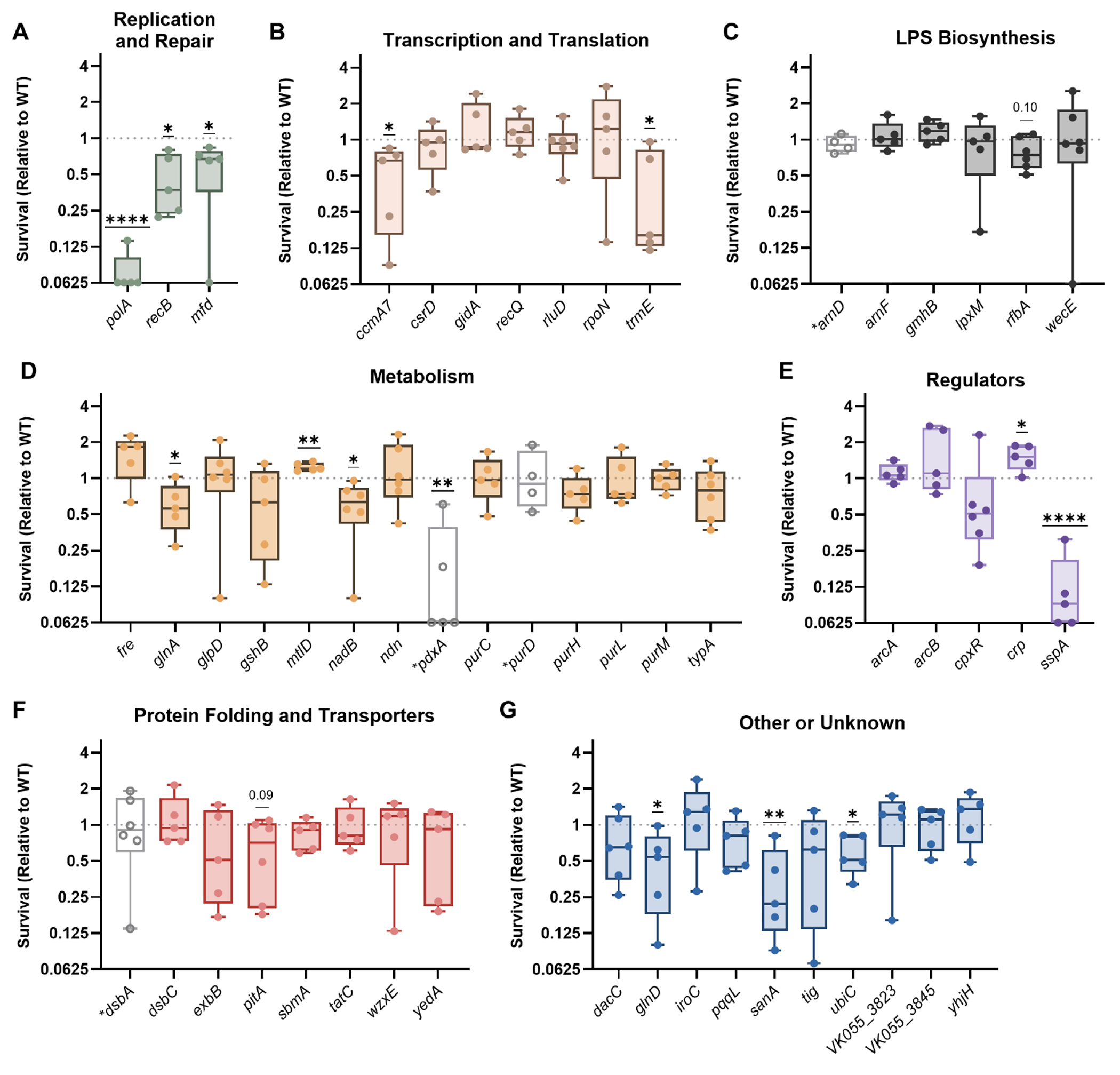
*K. pneumoniae* requires multiple factors to resist oxidative stress. Wild-type KPPR1 or transposon mutants with insertions in factors enhancing fitness in the spleen during bacteremia were exposed to 1 mM hydrogen peroxide for 2 h. Mutants were are displayed in groups by their predicted function, including (A) Replication and Repair, (B) Transcription and Translation, (C) LPS Biosynthesis, (D) Metabolism, (E) Regulators, (F) Protein Folding and Translocation, or (G) Other or Unknown functions. *K. pneumoniae* was quantified at the beginning (T0) and end (T2) of the experiment, and percent survival was calculated as (T2 CFU/T0 CFU) × 100. Survival (Relative to WT) was calculated by dividing the percent survival of each mutant by the percent survival of WT KPPR1 within the same assay. All experiments were performed in four to six independent trials; *P < 0.05, **P < 0.01, ***P < 0.001, and ****P < 0.0001 by a one-sample *t*-test with a hypothetical value of 1, *P* < 0.10 are indicated in text, and other comparisons were considered not significant. In all, *y*-axis data are displayed as a log_2_ fold change; box plot lines display the 25th, 50th, and 75th percentile values, whiskers indicate the minimum and maximum values, and points represent values from individual trials. Genes with hollow symbols and a lack of color indicate values previously published in reference 16 and reproduced here for comparison purposes.

After exposure to oxidative stress, 12/53 transposon mutants experienced a significant loss of fitness, and an additional two mutants trended toward a loss of fitness (Fig. 3A through G). All genes within the Replication and Repair category were required to resist oxidative stress, but genes involved in LPS biosynthesis and protein folding/transport were mostly distinct from protection against this threat (Fig. 3A, C, and F).

**FIG 4:**
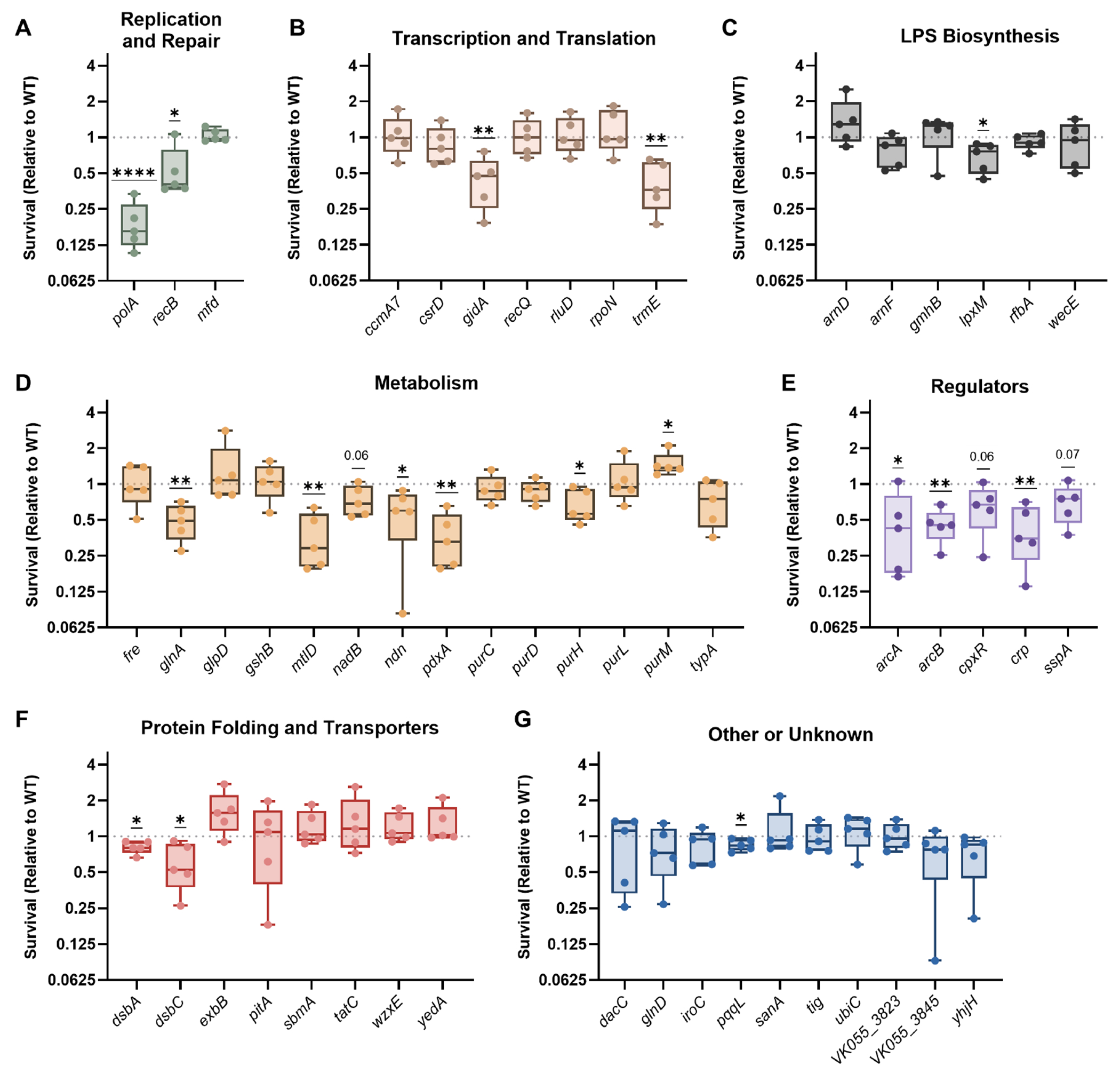
*K. pneumoniae* requires multiple factors to resist nitrosative stress. Wild-type KPPR1 or transposon mutants with insertions in factors enhancing fitness in the spleen during bacteremia were individually exposed to 25 mM DETA NONOate for 2 h. Mutants were are displayed in groups by their predicted function, including (A) Replication and Repair, (B) Transcription and Translation, (C) LPS Biosynthesis, (D) Metabolism, (E) Regulators, (F) Protein Folding and Translocation, or (G) Other or Unknown functions. *K. pneumoniae* was quantified at the beginning (T0) and end (T2) of the experiment, and percent survival was calculated as (T2 CFU/T0 CFU) × 100. Survival (Relative to WT) was calculated by dividing the percent survival of each mutant by the percent survival of WT KPPR1 within the same assay. All experiments were performed in five to six independent trials; *P < 0.05, **P < 0.01, ***P < 0.001, ****P < 0.0001 by a one-sample *t*-test with a hypothetical value of 1, *P* < 0.10 are indicated in text, and other comparisons were considered not significant. In all, *y*-axis data are displayed as a log_2_ fold change; box plot lines display the 25th, 50th, and 75th percentile values, whiskers indicate the minimum and maximum values, and points represent values from individual trials.

After exposure to nitrosative stress, 16/53 transposon mutants experienced a significant loss of fitness, with an additional three mutants trending toward lower fitness (Fig. 4A through G). Similar to oxidative stress resistance, genes involved in nitrosative stress resistance were linked to DNA replication and repair but largely distinct from LPS biosynthesis. Factors enhancing resistance to nitrosative stress were also not prevalent for genes within the Other or Unknown category. However, each regulator tested in this group of mutants had substantial fitness defects after exposure to DETA NONOate (Fig. 4E). The regulators in this group control complex networks, including the ArcAB and Crp metabolic pathways, the Cpx outer membrane stress response system, and the SspA stringent response regulator (32–34).

### Bacteremia fitness genes enhance resistance to macrophage-mediated, oxidative, and nitrosative stress in unique capacities

**FIG 5:**
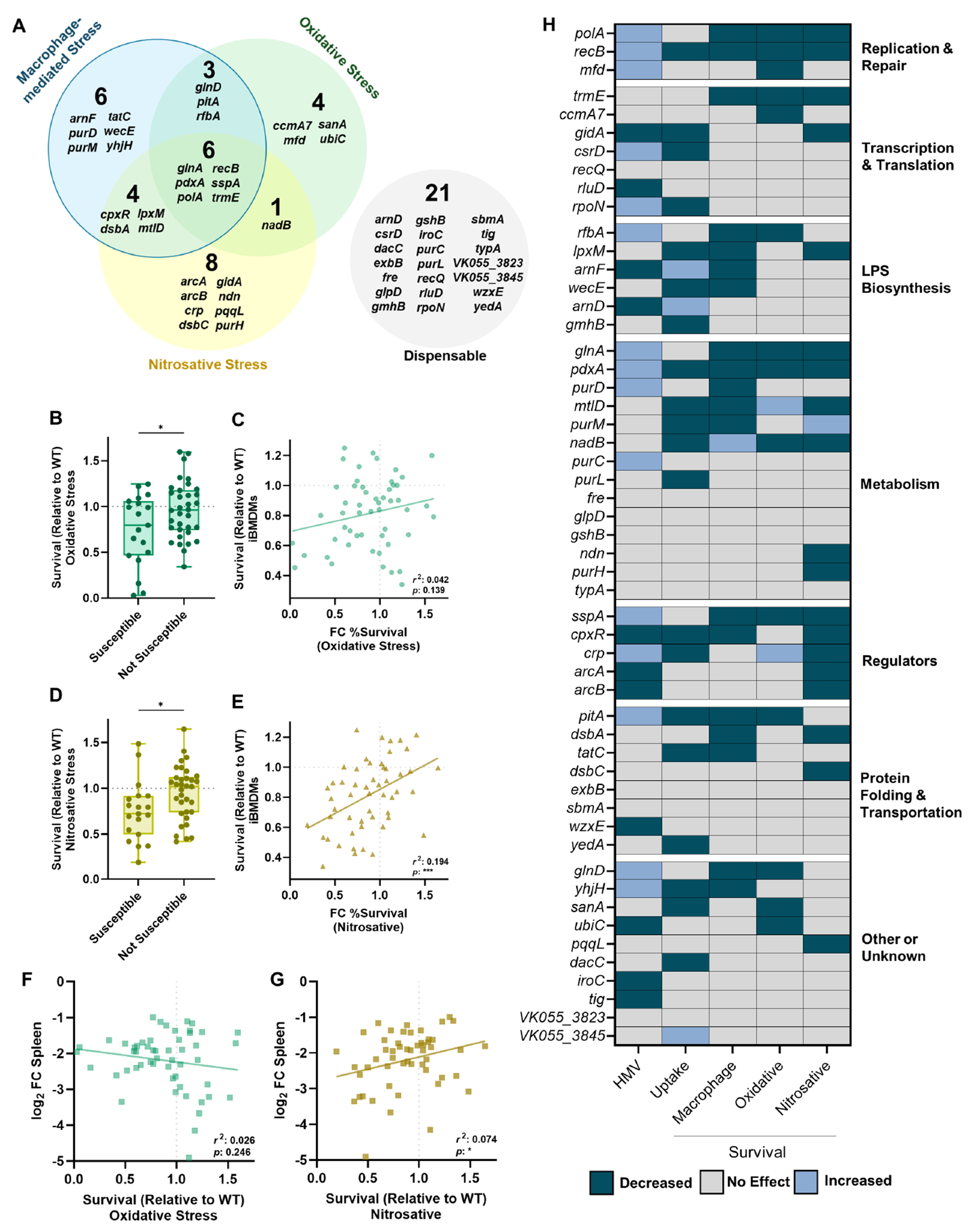
A summary of *K. pneumoniae* bacteremia fitness factors in the resistance to macrophage-mediated, oxidative, and nitrosative stress. (A) The distinct fitness factor contributions to macrophage-mediated, oxidative, and nitrosative stress resistance are displayed as a Venn diagram. (B–D) Mutants with intracellular fitness defects (Susceptible) were compared to those without survival defects (Not Susceptible) for their Survival (Relative to WT) in oxidative (B) and nitrosative (D) stress. The correlation between oxidative (C) or nitrosative (E) stress and intracellular survival, and between oxidative (F) or nitrosative (G) stress and spleen fitness *in vivo* is shown and assessed using a Pearson’s coefficient with *r*^2^ and *P*-values indicated on the graph. A heat map (H) summarizing whether the 53 transposon mutants assessed in this study had no effect (gray), increased (light blue), and decreased (teal) values for each type of assay. For (A and H), mutants were considered to influence survival and included within each category if their *P*-value was <0.10 in the screens within Fig. 1, 3 and 4. For (B–G), values are derived from the screen data in Fig. 1, 3 and 4. For (F and G), spleen fitness is derived from previously published transposon sequencing data comparing mutants in the gene at the output of infection compared to the input (16). In panels A, B, and D, mutants were considered to be “susceptible” to macrophage-mediated stress if they had a *P*-value < 0.10 in the Fig. 1 screen and “not susceptible” if the *P*-value was >0.10. For panels B and D, *P < 0.05 by unpaired *t*-test. Box plot lines display the 25th, 50th, and 75th percentile values, whiskers indicate the minimum and maximum values, and points represent values from individual trials.

Data collected in Fig. 1 to 4 reveal that a majority of *K. pneumoniae* bacteremia fitness factors are linked to stress resistance from common innate immune threats, as 60% of the tested genes enhanced resistance to macrophage-mediated, oxidative, or nitrosative stress. Overall, 36% of *K. pneumoniae* bacteremia fitness factors assisted with macrophage-mediated stress, 36% with nitrosative stress, and 26% with oxidative stress (Fig. 5A). Sorting these profiles further, we found that *K. pneumoniae* bacteremia fitness genes participate in stress resistance in multiple, often distinct, ways. For example, only six genes (*glnA, pdxA, polA, recB, sspA*, and *trmE*) were linked to resistance across all three stress conditions (Fig. 5A). In contrast, some genes were only relevant to one form of stress: four genes were solely linked to oxidative stress (*ccmA7, mfd, sanA*, and *ubiC*), eight genes to nitrosative stress (*arcA, arcB, crp, dsbC, gidA, ndn, pqqL*, and *purH*), and six genes to macrophage-mediated stress (*arnF, purD, purM, tatC, wecE*, and *yhjH*). In other cases, genes enhanced resistance to two stressors but were not involved in resistance to the third stressor. In the case of factors linked to macrophage-mediated stress, there were three genes linked to macrophage + oxidative stress (*glnD, pitA*, and *rfbA*) and four genes linked to macrophage + nitrosative stress (*cpxR, dsbA, lpxM*, and *mtlD*).

Next, we wanted to assess whether a mutant’s susceptibility to oxidative or nitrosative stress correlated with intracellular survival, as this may reveal strategies used by macrophages to kill *K. pneumoniae*. As a group, mutants with susceptibility to macrophage-mediated stress had significantly more susceptibility to oxidative stress compared to mutations that did not influence intracellular survival (Fig. 5B). However, there was not a significant correlation between oxidative stress survival and macrophage survival overall (Fig. 5C). In contrast, mutants that were categorized as susceptible to intracellular stress were significantly more susceptible to nitrosative stress (Fig. 5D), and in this case, the extent of survival after nitrosative stress exposure correlated with the degree of intracellular survival across all mutants (Fig. 5E). While these data demonstrate that *K. pneumoniae* resistance to oxidative and nitrosative stress is linked to increased intracellular survival, it implies that nitrosative stress resistance may be particularly relevant for *K. pneumoniae* fitness within monocyte-derived macrophages.

Using the percent survival values from the oxidative and nitrosative stress screens, we correlated each mutant’s ability to resist these stressors to their relative survival in the spleen during *in vivo* bacteremia (16). Although oxidative stress is important for host control of *K. pneumoniae* infection, survival in the presence of oxidative stress did not correlate with fitness in the spleen during infection (Fig. 5F). Instead, resistance to nitrosative stress significantly correlated with fitness in the spleen during bacteremia (Fig. 5G). Thus, macrophages may favor the elicitation of nitrosative stress to combat intracellular *K. pneumoniae*, which may be particularly relevant in the context of the spleen.

We also assessed whether *K. pneumoniae* elicits endogenous reactive oxygen species (ROS) and reactive nitrogen species (RNS) in response to oxidative and nitrosative stress. Wild-type KPPR1 was exposed to hydrogen peroxide or DETA NONOate, and bacterial ROS or RNS was assessed using the fluorescent probe DC-FDA or DAF-FM, respectively (Fig. S4A and B). In response to nitrosative stress, *K. pneumoniae* generated a significant ROS and RNS response. In contrast, under oxidative stress, *K. pneumoniae* did not generate either ROS or RNS. Thus, *K. pneumoniae* may be especially vulnerable to nitrosative stress in the intracellular compartment by generating endogenous reactive species in response to this threat. Sensitivity to external stressors did not necessarily predict the extent of endogenous generation of reactive species, as Δ*pdxA*, required for resistance to both oxidative and nitrosative stress, had similar levels of ROS and RNS generation as wild-type KPPR1 after exposure to DETA NONOate.

We wanted to visualize how different genetic functional categories were related to forms of stress resistance (Fig. 5H). A heatmap was generated in which mutants were categorized for lower (teal) or elevated (light blue) HMV or uptake and categorized for decreased (teal) or increased (light blue) stress resistance. Each factor related to DNA repair was required to resist oxidative stress (*polA, recB*, and *mfd*). Four of seven genes in the Transcription and Translation category were dispensable for resisting intracellular, oxidative, and nitrosative stress, indicating that these factors enhance bacteremia through functions independent from the stressors in this study. Genes involved in LPS biosynthesis were largely associated with resistance to macrophage-mediated stress but generally not required to resist oxidative or nitrosative stress. Of 14 genes, 8 genes involved in metabolism were important to resist at least one form of stress. Every gene within the Regulator category was linked to nitrosative stress resistance, indicating that *K. pneumoniae* possesses multiple mechanisms to combat this threat. The categories of “Protein Folding and Transportation” and “Other” contained genes enhancing resistance to each stressor, but no specific patterns were observed for these groups.

In order to validate a direct effect of mutations on the observed phenotypes, we examined the contributions of two genes to stress resistance, *pdxA* and *mtlD*. PdxA enhances *K. pneumoniae* fitness during bacteremic pneumonia (16) and was involved in stress resistance against all conditions in our study (Fig. 5A). MtlD is a member of the mannitol metabolism pathway and is important for bacteremia fitness (13, 35, 36). MtlD was required for intracellular survival and nitrosative stress but was dispensable under oxidative stress (Fig. 5A). Previously, we demonstrated that *pdxA* complementation can rescue Δ*pdxA* defects *in vitro* after oxidative stress (16). Here, we used the previously described Δ*pdxA* mutant, the mutant containing the empty vector (Δ*pdxA*_ev_), and the corresponding complemented strain (Δ*pdxA*_comp_), and generated a tn::*mtlD* trans-complemented strain (tn::*mtlD*_comp_) as well. In rich media and minimal media with mannitol as the carbon source, tn::*mtlD*_ev_ experienced a fitness defect that was restored upon *mtlD* complementation (Fig. S5A through D). Assessing the intracellular fitness of *pdxA* and *mtlD* empty vector and complemented strains was not possible as carriage of the pACYC vector led to substantial variation in interactions with macrophages (Fig. S5E). Under nitrosative stress, the pACYC vector did not influence KPPR1 fitness (Fig. S5F). Both Δ*pdxA*_ev_ and tn::*mtlD*_ev_ fitness defects were significantly alleviated with *pdxA* or *mtlD* complementation, respectively. Thus, PdxA and MtlD contribute to *K. pneumoniae* survival under nitrosative stress.

## DISCUSSION

In this study, we leveraged an existing data set of *K. pneumoniae* bacteremia fitness factors (16) to investigate the overlap between genes enhancing bloodstream infection and those influencing resistance to prominent types of stress elicited by innate immunity. Our data reveal that *K. pneumoniae* fitness factors are largely linked to macrophage-mediated, oxidative, and nitrosative stress and that these factors often contribute to stress resistance in distinct ways (Fig. 5).

Grouping the *K. pneumoniae* factors into broad functional categories helped to illuminate general bacterial responses that are important under different stress conditions (Fig. 5H). Factors within the DNA Replication and Repair category were important across all three forms of stress. This is not surprising since ROS and RNS, both generated by macrophages, can disrupt DNA stability (37). ROS directly damages DNA bases, induces double-strand breaks, and creates protein modifications that limit repair. RNS can disrupt DNA replication, inhibit bacterial respiration, and exacerbate damage done by ROS (37). LPS biosynthesis was particularly important for resistance to macrophage-mediated, but not oxidative or nitrosative, stress resistance. LPS can influence bacterial physiology by altering outer membrane permeability, vesicle formation, and susceptibility to antibiotics or antimicrobial peptides (38). Since mutations in LPS biosynthesis genes did not render *K. pneumoniae* more susceptible to oxidative and nitrosative stress consistently, this molecule may instead be most important for protecting against soluble peptides produced by macrophages in the intracellular environment in response to *K. pneumoniae*. Each factor in the Regulator category was linked to nitrosative stress resistance. The genes regulated by these systems are highly extensive, indicating that *K. pneumoniae* may require multiple systems to support fitness in the presence of RNS.

We also confirmed that HMV was not linked to intracellular survival (Fig. 2A). Because HMV is a function of the polysaccharide capsule, we assessed whether the capsule was required to resist intracellular stress. Indeed, two distinct acapsular *K. pneumoniae* strains had increased uptake but no detectable loss of fitness (Fig. 2B and C). Thus, the presence of the capsule does not necessarily convey an intracellular survival advantage. In fact, for our Δ*rfaH* and Δ*galU* mutants, a lack of capsule conveyed a survival advantage over wild-type KPPR1. While the mechanism for this finding is unknown, other studies have similarly reported that loss of capsule in KPPR1 does not influence intracellular survival in other cell lines (39). Further work must assess whether these patterns are consistent across *K. pneumoniae* strains and capsule types.

LPS molecules consist of three major regions: Lipid A, the Inner Core, and the O-antigen. Genes contributing to the biosynthesis of all three regions were present in the bacteremia fitness genes (Lipid A: *arnD, arnF,* and *lpxM*; Inner Core: *gmhB*; O-antigen: *rfbA* and *wecE)*. Members of the *arn* operon modify Lipid A through the addition of a 4-amino-4-deoxy-L-arabinose (L-Ara4N) moiety (40). The presence of L-Ara4N on Lipid A increases the overall positive charge of the bacterial surface, allowing for repulsion of positively charged antimicrobial peptides like polymyxin (38). Mutations in *arnD* and *arnF* led to significantly lower *K. pneumoniae* HMV (Fig. S3C), indicating that a positive surface charge relayed by L-Ara4N may be important for the retention of polysaccharide chains. Accordingly, the *arnD* and *arnF* mutants had significantly higher uptake than wild-type KPPR1, which was likely due to lower HMV or capsule. Neither ArnD nor ArnF were required for resistance to oxidative and nitrosative stress. This indicates that the L-Ara4N modification is not necessarily important for ROS and RNS resistance. Given this, it was intriguing that the *arnF* mutant had a significant intracellular fitness defect while *arnD* did not (Fig. 1C). In the L-Ara4N modification process, ArnD is a cytoplasmic enzyme that deformylates the L-Ara4N precursor prior to its transportation to the inner membrane. Once in the inner membrane, ArnF serves as a flippase to deliver the L-Ara4N precursor to ArnT, whose modification and export are the final steps of the process (40). Our data suggest that accumulation of L-Ara4N in the periplasm, in the absence of ArnF flippase activity, is more detrimental for bacterial fitness than when biosynthesis is halted in the cytoplasm in the absence of ArnD. Thus, the *arnF* intracellular fitness defect observed here is likely due to membrane stress and not Lipid A modification. Notably, *arnD* and *arnF* mutants were both significantly defective in the spleen during *in vivo* bacteremia, but *arnF* experienced nearly twice as great a defect as *arnD* (16). While Lipid A modifications are important for combating antimicrobial peptides *in vivo*, destabilization of the membrane in the *arnF* mutant likely imposes an additional stress on *K. pneumoniae*.

GmhB is important for spleen and liver fitness during bacteremia (11, 15), but its role in pathogenesis appears to be independent from the stressors investigated in our current study (Fig. 1C, 3C, and 4C). GmhB is involved in ADP-heptose biosynthesis, which is incorporated into the LPS inner core across Gram-negative species and is required to produce normal LPS in *K. pneumoniae* (15). Soluble ADP-heptose can also be detected within macrophage cytosol by the receptor ALPK1, leading to TIFAsome formation and inflammation (41–44). Normal LPS core structure protects bacteria from multiple types of antibiotics and likely also protects from soluble peptides produced by the host (45). Because a *gmhB* mutant has similar inflammatory profiles to wild-type KPPR1 *in vivo* and lack of GmhB does not influence resistance to oxidative or nitrosative stress, our data suggest that normal core structure protects *K. pneumoniae* from a stressor not detected in our current study, such as an extracellularly secreted soluble peptide. Alternatively, *gmhB* mutations do not render a complete loss of the *K. pneumoniae* LPS inner core (15). Therefore, it is possible that *K. pneumoniae* has redundant mechanisms for ADP-heptose production and that GmhB has additional, unrelated roles in pathogenesis that are not yet determined.

Many factors linked to metabolism are important for *K. pneumoniae* fitness during bacteremia, and about half of these genes (8/14) were linked to resistance against one of the three forms of stress (Fig. 5H). We were particularly interested in PdxA due to its previously observed *in vitro* and *in vivo* phenotypes. PdxA is involved in vitamin B_6_ biosynthesis and is a member of a complex multifunctional operon (17, 46). *In vitro,* PdxA protects *K. pneumoniae* from oxidative stress, but its role *in vivo* is more complicated. During infection, PdxA is required for *K. pneumoniae* fitness in the lung and spleen, yet this phenotype is also observed in mice lacking phagocyte ROS production. Therefore, PdxA likely provides protection against ROS and other forms of stress during infection (16). Here, we further determined that PdxA protects *K. pneumoniae* against nitrosative stress. Since our data demonstrate that nitrosative stress survival is correlated to macrophage survival and spleen fitness *in vivo* (Fig. 5), PdxA may protect against RNS during infection. *Enterobacterales* possess multiple mechanisms for vitamin B_6_ acquisition, including pathways for endogenous biosynthesis and scavenging from the environment (46). Therefore, it is likely that PdxA has undescribed roles in pathogenesis beyond vitamin B_6_ metabolism.

In our present study, MtlD was important for intracellular fitness and resistance to nitrosative stress but was dispensable for oxidative stress. MtlD is a member of the mannitol metabolism pathway and converts mannitol-1-phosphate to fructose-6-phosphate, which can then be utilized for glycolysis. The identification of a mannitol metabolism gene in our initial transposon sequencing screen was curious since the abundance of mannitol in mammalian serum is low (47). However, MtlD has been identified as a bacteremia fitness factor in multiple Gram-negative species (36, 48). As such, MtlD likely protects *K. pneumoniae* from stress through a mechanism independent from the utilization of mannitol as a carbon source in the bloodstream. In *Salmonella*, MtlD has been proposed as a therapeutic target, as bacteria lacking *mtlD* experience sugar phosphate toxicity in the presence of mannitol (35, 36). As LB contains trace amounts of mannitol, it is possible that our *mtlD* mutant was exhibiting low levels of sugar phosphate toxicity at the beginning of each stress assay and was, therefore, more vulnerable to macrophages and NONOate. This may indicate that oxidative stress does not exacerbate sugar phosphate toxicity as does nitrosative stress.

Oxidative stress resistance is relevant to *K. pneumoniae* pathogenesis in multiple studies (11, 16, 49), so we were surprised to find bacterial mutations that led to higher survival in the presence of this stress. Cyclic-AMP receptor protein (CRP) is a global regulator that controls transcription of multiple bacterial operons and genes (33). A major role for CRP is to shift bacterial metabolic activity to preferred carbon sources, like glucose, based on availability within the environment (50). The *crp* mutant displayed increased HMV, although these values were highly variable (Fig. S3E). Therefore, altered carbon metabolism likely influences the efficiency of capsule biosynthesis. This, in turn, affects uptake by macrophages, as the *crp* mutant was significantly resistant to internalization. Because we detected no intracellular fitness defects for the *crp* mutant, the level of nitrosative stress encountered within the cell may not have reached the threshold observed in our *in vitro* assays. However, we did detect substantial differences in interactions with oxidative and nitrosative stress. Mutations in *crp* led to significantly greater protection against oxidative stress and defects against nitrosative stress. Since the CRP regulatory network is extensive (33, 51), it is possible that mutations in *crp* prevent *K. pneumoniae* from shifting to a metabolic pathway that is unfavorable during oxidative stress. This also indicates that *K. pneumoniae* resistance to oxidative and nitrosative stress may involve different metabolic pathways. Little is known about the intracellular lifestyle of *K. pneumoniae,* so it is unclear which pathways may be initiated under these stress conditions.

The present study focused on the role of bacterial factors that enhance *K. pneumoniae* fitness within macrophages, as few factors enhancing intracellular survival have been described for this species. However, it should be noted that neutrophils are also highly recruited during infection and play a significant role in *K. pneumoniae* clearance (22, 49, 52). As neutrophils produce a stronger ROS burst than macrophages (53), it is likely that the Venn diagram in Fig. 5A would shift if neutrophils were used instead. In this scenario, we would expect that more of the oxidative stress hits would overlap with factors that enhance bacterial survival in the presence of neutrophils. Given the significant role of neutrophils during *K. pneumoniae* infection, we believe that many of the bacteremia fitness hits investigated in this study would also enhance resistance to clearance by neutrophils. Future studies should further investigate the interactions between *K. pneumoniae* and multiple immune subsets, including neutrophils. While the focus of this study was to understand bacterial stress resistance during bacteremia, we can also learn much about host-pathogen interactions by deciphering these patterns. For example, to enhance intracellular survival, *K. pneumoniae* leverages some genes that are involved in either oxidative or nitrosative stress, some genes that are involved in resisting both, and some genes that are involved in neither. Thus, *K. pneumoniae* defense against macrophages is multifaceted and requires strategies to combat distinct threats. We are particularly interested in the significant links revealed between nitrosative stress, intracellular stress, and bacteremia (Fig. 5D, E and G). While oxidative stress is indeed important for host resistance to *K. pneumoniae* infection, our data reveal that resistance to nitrosative stress may be particularly important in the context of the macrophage intracellular environment and in the context of the spleen. Further supporting this finding, our previous data show that loss of ROS production in the lung leads to significantly higher *K. pneumoniae* bacterial burden, but that loss of ROS in the spleen has a subtle effect on colonization (11). Combined, we hypothesize that the spleen skews toward nitrosative stress mechanisms during bacteremia. Major pathways used by macrophages to kill *K. pneumoniae* remain unresolved and will be the focus of future studies.

Limitations of our study include the use of a single *K. pneumoniae* strain and the use of a single macrophage subset. *K. pneumoniae* is a remarkably diverse bacterial strain, and different pathotypes may employ unique strategies to perpetuate infection. While this is likely the case for a few factors, many of these genes identified in our study are highly conserved and likely applicable to many strains and bacterial species. It is also appreciated that different subsets of macrophages have unique interactions with pathogens. Our study focused on monocyte-derived macrophages, as these are present during *K. pneumoniae* infections across tissues and are also available in high yields to perform screens on multiple mutants. However, niche-specific microenvironments that include the presence of different tissue-resident macrophages may influence these fitness patterns across sites *in vivo*. The macrophages used in our study were naive, yet demonstrated significant levels of *K. pneumoniae* killing over a 4-h period. While this was sufficient for the present study, priming macrophages to an M1 or M2 state may also influence the patterns of intracellular killing that are observed.

Together, our study characterized a toolbox of fitness factors used by *K. pneumoniae* to enhance bacteremia. We describe the large overlap in factors enhancing bacteremia with factors enhancing resistance to macrophage-mediated, oxidative, and nitrosative stress. These data highlight how *K. pneumoniae* uses diverse strategies to enhance resistance to multiple forms of stress.

## MATERIALS AND METHODS

### Bacterial strains and materials

All materials and reagents were sourced from Sigma-Aldrich (St. Louis, MO, USA) unless stated otherwise. *K. pneumoniae* strains were cultured in LB broth (Fisher Bioreagents, Ottawa, ON, Canada) overnight for roughly 14–16 h, shaking at 37°C prior to use in experiments. *K. pneumoniae* was also plated onto LB agar (Fisher Bioreagents) and incubated on plates overnight at 37°C. When culturing isogenic knockout or transposon strains, media were supplemented with 40 μg/mL kanamycin. When culturing strains carrying the pACYC184 plasmid, media were supplemented with 50 μg/mL chloramphenicol. Table S2 details the strains used in this study. For all experiments using pACYC, strains were generated from a single transformation and then recovered from freezer stocks onto agar dishes prior to starting overnight cultures for subsequent use.

All transposon mutants were selected from a previously described KPPR1 arrayed library (24) and insertions verified by primers flanking the gene of interest. Primers used for this study are in Table S3. The KPPR1 strain with constitutive chromosomal expression of GFP (KPPR1-chromoGFP) was generated for this study using Lambda red mutagenesis as previously detailed (54). In short, a small intergenic region was removed between base pairs 2,306,546 and 2,306,610 of the KPPR1 genome (55) and replaced with a cassette from the pIDMv5k plasmid containing DasherGFP and a kanamycin resistance cassette under constitutive expression by the J23100 promoter. To generate mutants, electrocompetent cells were prepared by culturing KPPR1 carrying the pKD46 plasmid at 30°C in media containing 50 μg/mL spectinomycin overnight. The bacteria were diluted 1:50 in media containing 50 μg/mL spectinomycin, 50 mM L-arabinose, 0.5 mM EDTA (Promega, Madison, WI, USA), and 10 μM salicylic acid until the exponential phase was reached. Bacteria were placed on ice for 30 min and then serially washed using centrifugation at 8,000 x *g* for 15 min at 4°C using the following steps: (i) 50 mL of 1 mM HEPES (pH 7.4; Gibco, Grand Island, NY, USA), (ii) 50 mL diH_2_O, and (iii) 20 mL 10% glycerol in diH_2_O. The final suspension was adjusted to 2–3 × 10^10^ in 10% glycerol. The promoter-GFP-kanamycin cassette fragment was electroporated into electrocompetent KPPR1, and transformants were selected at 37°C on LB agar containing kanamycin. To generate the tn::*mtlD* complement strain, electrocompetent tn::*mtlD* cells were generated as above. Gibson assembly was used to create pACYC*_mtlD_* with NEBuilder HiFi DNA Assembly Master Mix (New England Biolabs, Ipswich, MA, USA). pACYC184 (the empty vector, or pACYC_ev_) was linearized using BamHI and HindIII (New England Biolabs). The *mtlD* region, plus the region upstream containing promoter sites (predicted by SoftBerry BPROM; Softberry Inc., Mount Kisco, NY, USA), was mixed with the linearized pACYC and incubated with the Gibson Assembly Mix per the manufacturer’s instructions. The resulting product was maintained in *Escherichia coli* TOP10 cells (New England Biolabs) and confirmed to be pACYC*mtlD* using sequencing (Plasmidsaurus, San Francisco, CA, USA) and internal primers for *mtlD*. Then, the empty pACYC plasmid (pACYC_ev_) or the complement vector (*pACYC_mtlD_*) was mobilized into electrocompetent tn::*mtlD*.

### Cell culture and gentamicin protection assay

Immortalized bone marrow-derived macrophages initially derived from C57BL/6J mice were used for each assay in this study (56, 57). To culture macrophages, cells were maintained in DMEM + 10% FBS + 1% penicillin-streptomycin and passaged when at ~75% confluency. To passage macrophages, cells were lifted from tissue-culture-treated plastic with 2 mM ice-cold EDTA and gentle washing. Cells were then pelleted at 500 × *g* for 5 min at 4°C, and resuspended cells were quantified by counting using a hemacytometer. Trypan exclusion was used during counting to assess cell viability, and macrophages were not passaged if they demonstrated viability <90%. Cells in this study were used between passages 2 and 12, as we observed decreased killing capacity at passages greater than 15. To perform experiments, macrophages were seeded at 1 × 10^6^ cells per well in DMEM + 10% FBS and infected with 1 × 10^7^ WT KPPR1 or each of the TnSeq mutants (MOI 10). Plates were incubated at 500 × *g* for 5 min to facilitate contact and incubated for 1 h at 37°C and 5% CO_2_. Cells were then treated with 120 μg/mL gentamicin for 30 min to kill extracellular bacteria. Afterward, a subset of cells was washed with PBS and immediately lysed with 1× filter-sterilized saponin and plated to enumerate intracellular *K. pneumoniae* at T0, or the uptake. A separate subset of wells was incubated for an additional 4 h prior to washing and lysis, then plated to quantify intracellular survival of *K. pneumoniae* at T4, or the endpoint (output). The percent survival of each strain was calculated as (CFU T4/CFU T0) × 100. This allowed us to compare killing across strains while controlling for variation in uptake. Each trial was run in parallel with wild-type KPPR1, and a fold-change percent survival was generated that compared each mutant strain to the wild type. This allowed for normalization across trials and batches of cells.

To assess uptake, bone-marrow-derived macrophages (BMDMs) were infected with a single mutant for 1 h, treated with gentamicin, then lysed to quantify intracellular *K. pneumoniae*. A separate well was incubated for an additional 4 h after gentamicin treatment, then lysed to quantify intracellular survival. The percent survival of each strain was calculated by dividing the bacterial abundance at the endpoint by the abundance at uptake.

### HMV assay

HMV was assessed by a centrifugation assay as previously described (16). Cultures of *K. pneumoniae* strains were grown overnight in 1.5 mL LB, and 500 μL of the overnight culture was added to 1.5 mL PBS and mixed. The OD_600_ of the suspension was measured by removing 900 μL of the sample (pre-spin value). The remaining suspension was centrifuged at 1,000 × *g* for 5 min, the top 900 μL of the sample was removed, and the OD_600_ was measured (post-spin). Percent HMV was calculated as (OD_600_ post-spin)/ (OD_600_ pre-spin) × 100 (24).

### Oxidative stress assay

WT KPPR1 and each of the TnSeq mutants were exposed to hydrogen peroxide to assess resistance to oxidative stress as previously described (16). CFUs (1 × 10^6^) of each strain were exposed to 1 mM hydrogen peroxide for 2 h at 37°C, and *K. pneumoniae* was quantified at the beginning (T0) and end (T2) of the experiment. Percent survival was calculated as (CFU T2)/(CFU/T0) × 100. The percent survival of each mutant was normalized to the killing of KPPR1 within the same assay to generate a fold change percent survival.

### Nitrosative stress assay

WT KPPR1 and each of the TnSeq mutants were exposed to DETA NONOate to assess resistance to nitrosative stress (52). CFUs (1 × 10^6^ ) of each strain were exposed to 25 mM DETA NONOate for 2 h at 37°C, and *K. pneumoniae* was quantified at the beginning (T0) and end (T2) of the experiment. Percent survival was calculated as (CFU T2)/(CFU/T0) × 100. The percent survival of each mutant was normalized to the killing of KPPR1 within the same assay to generate a fold change percent survival.

### Growth curves

Overnight cultures of *K. pneumoniae* were adjusted to 1 × 10^7^ CFU/mL in LB broth or M9 salts (Gibco) containing 0.4% mannitol. An Eon microplate reader with Gen5 software (Version 2.0, BioTek, Winooski, VT, USA) measured OD_600_ every 15 min. Plates were incubated at 37°C for the duration of the experiment with orbital shaking before each read. Differences in growth were detected by measuring the area under the curve (GraphPad Prism Software, La Jolla, CA, USA).

### Assessment of bacterial ROS and RNS

A total of 1 × 10^7^ CFUs of the strains KPPR1 and Δ*pdxA* were exposed to 1 mM hydrogen peroxide or 25 mM DETA NONOate for 1 h. Bacterial suspensions were then pelleted, washed, and resuspended in fluorescent probe and measured in a plate reader as instructed by the manufacturer. The generation of ROS and RNS was determined using the fluorescent probes DC-FDA (13 μM; Cayman Chemicals, Ann Arbor, MI, USA) and DAF-FM diacetate (5 μM; ThermoFisher), respectively. For each condition, viable bacterial counts were assessed after treatment.

### Statistical analysis

Statistical significance was evaluated using GraphPad Prism software and defined as a *P*-value < 0.05. This was determined using a one-sample test to assess differences between sample values and a hypothetical value of 1 for assessments of macrophage uptake, intracellular survival, oxidative stress, and nitrosative stress, unpaired *t*-tests where indicated assess differences between two groups. Each *in vitro* assay was performed in at least four biological replicates.

## Supplementary Material

Supplemental Table 2 **Table S2 (IAI00739-25-s0003.xlsx).** Strains used in this study.

Supplemental Table 3 **Table S3 (IAI00739-25-s0004.xlsx).** Primers used in this study.

Supplemental Table 1 **Table S1 (IAI00739-25-s0002.xlsx).** CFU values from the three screens in this study.

Supplemental Material
