# Supplementary material for: Klebsiella pneumoniae factors enhancing bacteremia have distinct contributions to macrophage-mediated, oxidative, and nitrosative stress resistance
Source: Infect Immun. Author manuscript; Available in PMC 2026 Apr 18. (PMC13081721; doi:10.1128/iai.00739-25)
Supplement: Supplemental Material [file NIHMS2159215-supplement-Supplemental_Material.docx]

**Supplemental Material**


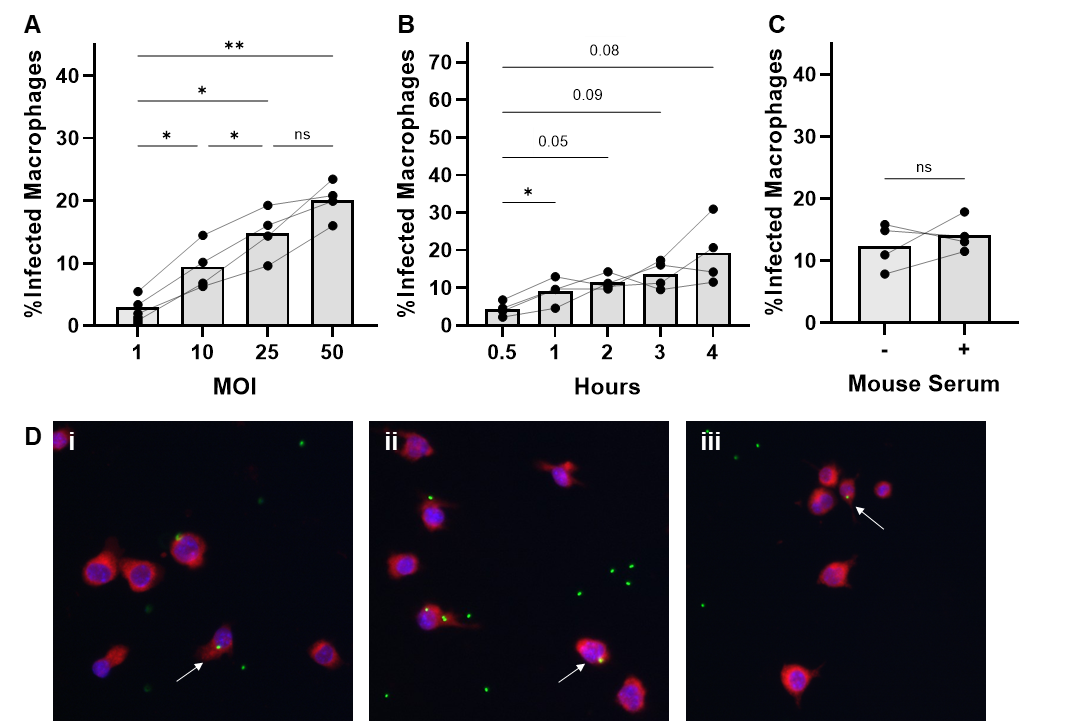


***Supplemental Figure 1.* Optimization of gentamicin protection assays using KPPR1.** To optimize interactions between *K. pneumoniae* strain KPPR1 and BMDMs, the proportion of macrophages either associated or unassociated with KPPR1-chromoGFP were assessed by fluorescent microscopy. (A) The ratio of bacteria to macrophages (multiplicity of infection, MOI), (B) time of infection, (C) and inferred opsonization after KPPR1 incubation with mouse serum were assessed for contributions to uptake. For all, percent infected macrophages was calculated as: (number of GFP^+^ macrophages/number of total macrophages)*100. All experiments were performed in 4 independent trials; **p*<0.05, ***p*<0.01 by (A) one-way ANOVA with Tukey’s correction comparing all groups to each other, (B) one-way ANOVA with Dunnet’s correction comparing each group to uptake at 30 minutes, and (C) paired *t-*test. In all, bar graph height represents the mean values for each group. Three example images (Di-iii) for experiments in A-C are displayed to demonstrate cells that were classified as associated with KPPR1-chromoGFP (indicated by the white arrows). Example images were taken from cells infected at an MOI 10 after (Di) 30 minutes, (Dii) 1 hour, or (Diii) 3 hours of contact time with unopsonized bacteria. For fluorescence microscopy, macrophages were infected as described in the Materials and Methods for gentamicin protection assays. However, instead of lysing cells at T4, cells were washed and fixed with 100µL 4% paraformaldehyde in PBS for 30 minutes at 4°C. Cells were then stained with 1x CellTracker Red and 1x DAPI, and washed three times before visualization. A *Kp* associated macrophage was defined as a cell in which green bacteria was within the bounds the red macrophage cell border.

**
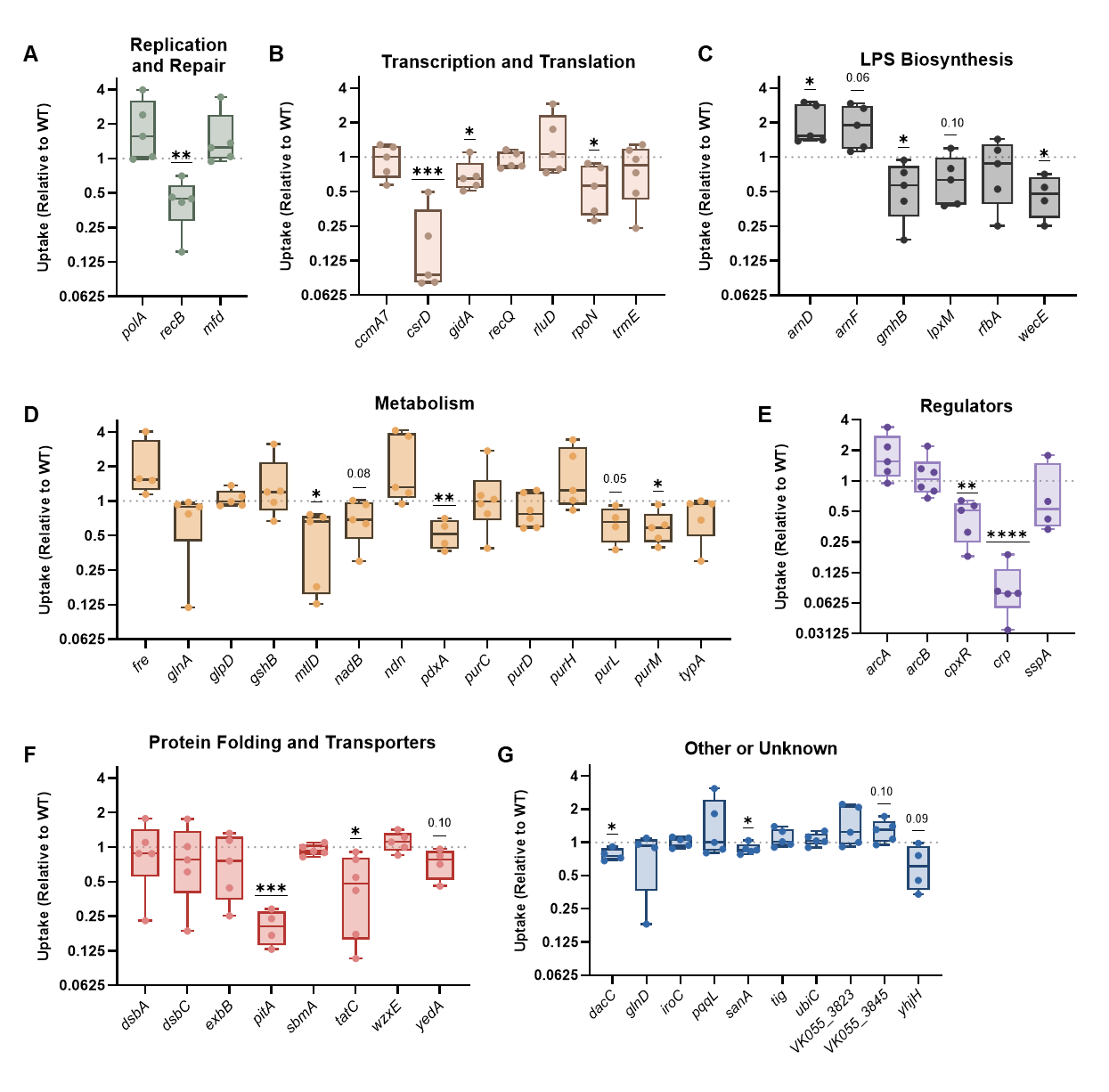
**

***Supplemental Figure 2.* Multiple bacteremia fitness factors influence *K. pneumoniae* uptake by bone marrow-derived macrophages.** Bone-marrow derived macrophages were infected with wild-type KPPR1 or transposon mutants with insertions in factors enhancing fitness in the spleen during bacteremia. Mutants were are displayed in groups by their predicted function, including (A) Replication and Repair, (B) Transcription and Translation, (C) LPS Biosynthesis, (D) Metabolism, (E) Regulators, (F) Protein Folding and Translocation, or (G) Other or Unknown functions. After 1 hour of infection, extracellular bacteria were killed with gentamicin treatment. Cells were then lysed and intracellular *K. pneumoniae* enumerated to quantify baseline bacterial uptake (T0) as CFU/mL. Then, the uptake of each mutant was normalized to WT KPPR1 within the same assay to generate a fold change uptake. All experiments were performed in 4-6 independent trials; **p*<0.05, ***p*<0.01, ****p*<0.001, *****p*<0.0001 by a one-sample *t-*test with a hypothetical value of 1; *p<*0.10 are indicated in text and other comparisons were considered not significant. In all, y-axis data is displayed as a log_2_ fold change; box plot lines display the 25^th^, 50^th^, and 75^th^ percentile values, whiskers indicate the minimum and maximum values, and points represent values from individual trials.

***Supplemental Figure 3.* HMV values for *K. pneumoniae* bacteremia fitness factors.** The extent of hypermucoviscosity (HMV) for transposon mutants with insertions in factors linked to enhancing splenic fitness during bacteremia were assessed by a centrifugation buoyancy assay. Mutants were are displayed in groups by their predicted function, including (A) Replication and Repair, (B) Transcription and Translation, (C) LPS Biosynthesis, (D) Metabolism, (E) Regulators, (F) Protein Folding and Translocation, or (G) Other or Unknown functions. %HMV was calculated as: (OD_600_ post-centrifugation)/(OD_600_ pre-centrifugation)*100. Each assay was run in parallel with wild-type KPPR1 and positive and negative controls known to have high and low HMV, respectively. All experiments were performed in 4-5 independent trials; **p*<0.05, ***p*<0.01, ****p*<0.001, *****p*<0.0001 by a one-sample *t-*test with a hypothetical value of 11.33 (the average wild-type KPPR1 %HMV for these assays), *p<*0.10 are indicated in text and other comparisons were considered not significant. In all, box plot lines display the 25^th^, 50^th^, and 75^th^ percentile values, whiskers indicate the minimum and maximum values, and points represent values from individual trials. The dotted line represents the average wild-type KPPR1 %HMV value across all trials. Genes with hollow symbols and lack of color indicate values previously published in (*16*) and reproduced here for comparison purposes.


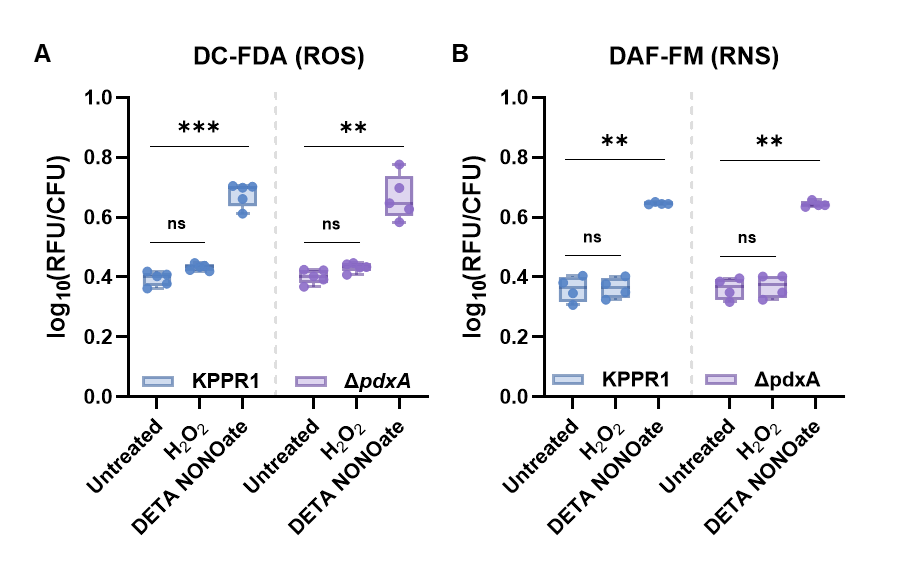


***Supplemental Figure 4. K. pneumoniae* generation of ROS and RNS in response to stress.** The extent to which *K. pneumoniae* generates endogenous (A) ROS and (B) RNS in response to oxidative and nitrosative stress was assessed. The strains KPPR1 and Δ*pdxA* were exposed to hydrogen peroxide or DETA NONOate. The generation of ROS and RNS within bacterial suspensions was determined using the fluorescent probes (A) DC-FDA and (B) DAF-FM diacetate. For each condition, viable bacterial counts were assessed after treatment and data is visualized as log_10_(relative fluorescent units/colony forming units). For each, ***p<*0.01, ****p<*0.001, by one-way ANOVA comparing untreated to treated bacteria, and comparisons were evaluated separately for each strain. For A and B, the signal between DETA NONOate treated KPPR1 and Δ*pdxA* was not significant as assessed by a paired *t-*test. In all, box plot lines display the 25^th^, 50^th^, and 75^th^ percentile values, whiskers indicate the minimum and maximum values, and points represent values from individual trials.


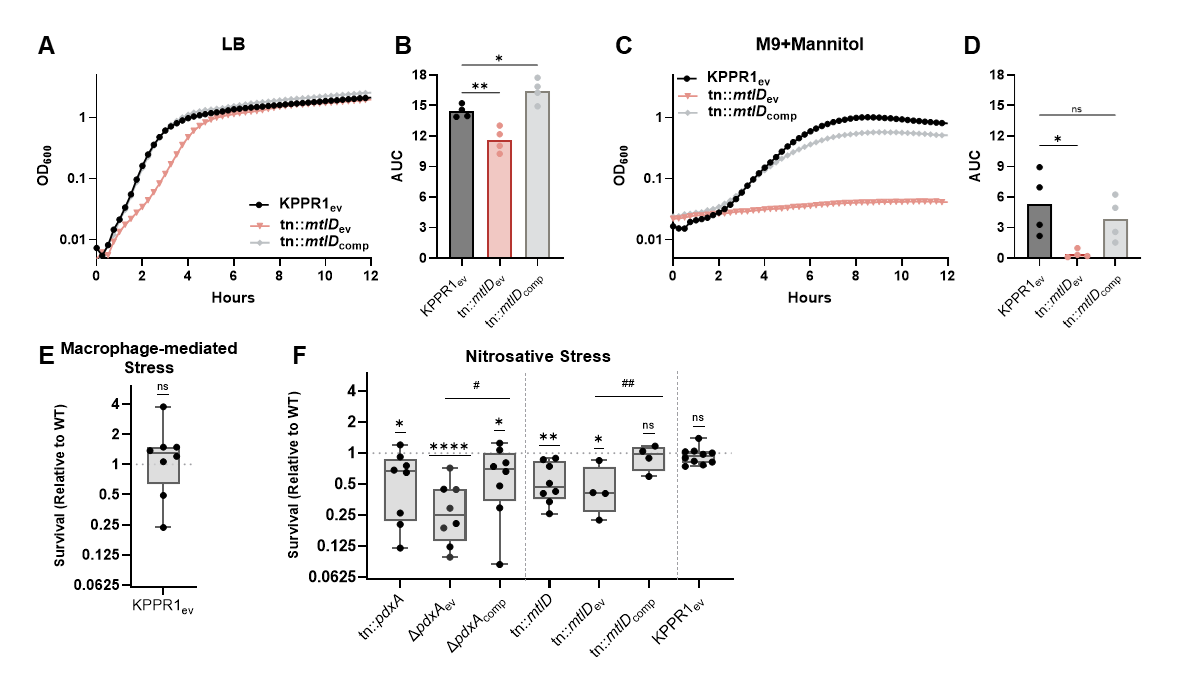


***Supplemental Figure 5.* PdxA and MtlD contribute to *K. pneumoniae* stress resistance.** KPPR1 or tn::*mtlD* carrying the empty pACYC plasmid (empty vector, ev) or tn::*mtlD* carrying pACYC with *mtlD* expression (comp) were grown in (A-B) LB or (C-D) M9+Mannitol. (A, C) Growth curves represent the average OD_600_ values taken every 15 minutes from cumulative trials and (B, D) area under the curve (AUC) analysis represents cumulative growth based on the final growth curve. In (A-D), n=4 independent trials; in (B, D) **p<0.05, **p<0.01* by paired one-way ANOVA comparing tn::*mtlD* strains to KPPR1. Points represent values from individual trials and bars represent mean values. (E) Gentamicin protection assays demonstrated that carriage of the pACYC vector leads to high variability in intracellular survival compared to KPPR1 not carrying pACYC. (F) The tn::*pdxA* and tn::*mtlD* results from the original screens in Figures 2 and 5 were re-validated and strains were run alongside mutants carrying either the empty pACYC vector (ev) or the pACYC carrying either *pdxA* and *mtlD* expression (complement strains; comp). The mutants, empty vector, and complement strains were assessed in nitrosative stress assays. In (A-F), all experiments were performed in 4-10 independent trials. In (F), **p*<0.05, ***p*<0.01, *****p*<0.0001 by a one-sample *t-*test with a hypothetical value of 0; ^#^*p*<0.05, ^##^*p*<0.01 by paired *t-*test. In E-F, y-axis data is displayed as a log_2_ fold change; box plot lines display the 25^th^, 50^th^, and 75^th^ percentile values, whiskers indicate the minimum and maximum values, and points represent values from individual trials.

***Supplemental Table 1.*** Values and output for the macrophage-mediated, oxidative, and nitrosative stress screens. For macrophage-mediated stress, the input values are the CFU added to each well and used to generate the MOI (Input/Number of macrophages in the well). T0 is the abundance of CFU detected intracellularly after 1 hour of incubation time between macrophages and *K. pneumoniae*. Percent uptake is calculated as (T0 CFU/Input CFU)*100. T4 is the abundance of CFU detected intracellularly after 4 hours of incubation time between macrophages and *K. pneumoniae*. Percent survival is calculated as (T4 CFU/T0 CFU)*100. For oxidative and nitrosative stress, T0 is the abundance of CFU present in the well at the beginning of the assay. T2 is the abundance of CFU present in the well after incubation with hydrogen peroxide or DETA NONOate. Percent survival is calculated as (T2 CFU/T0 CFU)*100.

***Supplemental Table 2.*** Bacterial strains used in this study.

***Supplemental Table 3.*** Primers used in this study.
